# Various Congenital Abnormalities and Anatomic Variants of the Pancreas: A Pictorial Review

**DOI:** 10.5334/jbsr.1780

**Published:** 2019-06-28

**Authors:** Seung Soo Kim, Hyeong Cheol Shin, Jeong Ah Hwang

**Affiliations:** 1 Soonchunhyang University College of Medicine, Cheonan Hospital, KR

**Keywords:** Pancreas, Congenital abnormalities, Anatomic variation, Multidetector computed tomography, Magnetic resonance imaging

## Abstract

Numerous and various congenital abnormalities and anatomic variants of the pancreas (CAAVPs) have been reported. Some of them are not so uncommon. Recent advances and accessibility of various multiplanar imaging modalities today offer the increased capabilities of detection and full diagnosis of these CAAVPs. With a precise diagnosis, the symptomatic CAAVPs can not only be more specifically treated but even more their detection and exact description can modify the surgical or interventional strategy to avoid unexpected post-operative complications. This article aimed to review the embryogenesis of the pancreas and describe imaging findings of CAAVPs.

## Introduction

Congenital abnormalities and anatomic variants of the pancreas (CAAVPs) are not uncommon and appear in a variety of ways [1]. Although many CAAVPs are incidentally detected due to the increasing accessibility of diagnostic imaging, some of them may present with symptoms, ranging from abdominal pain to pancreatitis [2]. It is important to recognize symptomatic CAAVPs because they can be corrected by surgery or interventional procedure [1,3]. In addition, CAAVPs can affect surgical planning [4]. Therefore, radiologists must be familiar with CAAVPs to correctly diagnose them.

## Imaging techniques

Computed tomography (CT) is relatively inexpensive and accessible, and preferentially used to evaluate the pancreas. Contrast-enhanced CT for the pancreas is performed 35–45 sec (pancreatic phase) and 60–70 sec (portal venous phase) after the start of IV injection of contrast material and uses thin sections for detailed characterization. The pancreatic parenchyma shows peak enhancement on the pancreatic phase [5]. Magnetic resonance imaging (MRI) reveals a clearer contrast to the soft tissue than CT. In particular, the pancreatic tissue shows a characteristic high signal intensity on the fat suppressed T1-weighted image [6]. In addition, MRI is more valuable for imaging children because of the absence of radiation exposure. Magnetic resonance cholangiopancreatography (MRCP) is useful for evaluation of the pancreatic duct.

## Embryonic development of the pancreas

**Figure 1:**
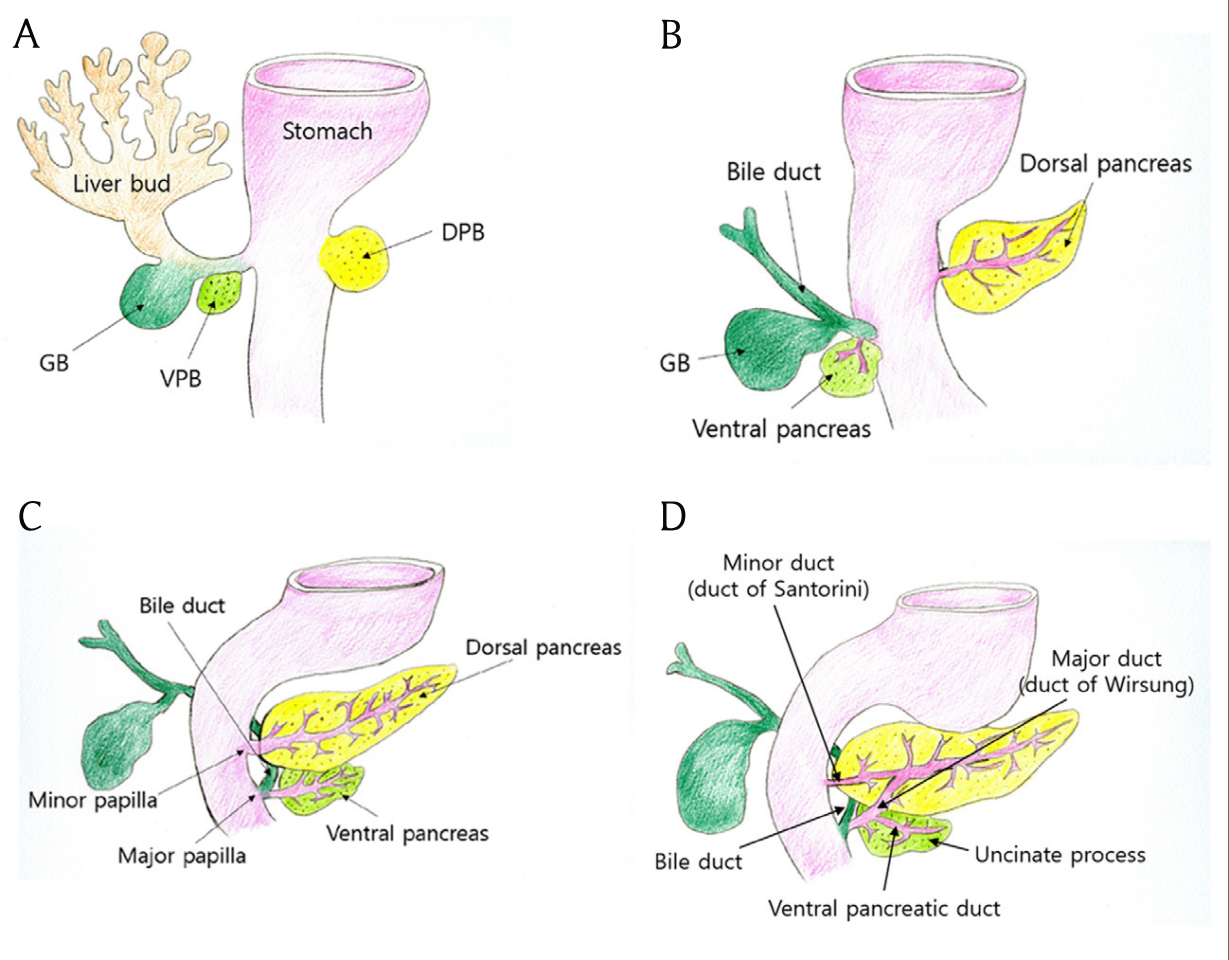
Illustration of the normal development of the pancreas. **(A, B)** Dorsal pancreatic bud develops from dorsal outpouching and ventral pancreatic bud is derived from the ventral outpouching. Liver, gallbladder, and bile duct also arise from the ventral outpouching. **(C, D)** As duodenum rotates, ventral pancreas moves behind duodenum. Dorsal pancreas becomes the upper part of head, body, and tail portion of the pancreas. In contrast, uncinate process and the lower part of head arise from the ventral pancreas. Finally, dorsal and ventral pancreatic ducts fuse. The ventral pancreatic duct becomes major duct (duct of Wirsung), which opens to the major papilla with the common bile duct. The small dorsal pancreatic duct (duct of Santorini) drains through the minor papilla. *DPB* dorsal pancreatic bud, *VPB* ventral pancreatic bud, *GB* gallbladder.

In the fourth week of the embryonic period, ventral and dorsal outpouchings arise at the junction of the foregut and midgut. The dorsal pancreatic bud arises from the dorsal outpouching, whereas the ventral pancreatic bud develops from the ventral one, which is also the primordium of the liver and biliary system (Figure 1A and 1B) [3,7]. As the stomach rotates, the duodenum rotates to the right and becomes C-shaped. Then, the ventral pancreas moves backwards and lies beneath and behind the dorsal pancreas, eventually merging with the dorsal pancreas at 37 fetal days [2,7]. The ventral pancreas becomes the uncinate process and the lower part of the pancreatic head, and the remainder of the pancreas is derived from the dorsal pancreas (Figure 1C). After fusion, ducts in the dorsal and ventral pancreas meet to form the major duct (duct of Wirsung) [2,7]. The major duct opens to the duodenum via the major papilla with the common bile duct. The duct in the upper head portion arising from the dorsal pancreas becomes the duct of Santorini, opening through the minor papilla to the duodenum (Figure 1D) [3].

## Congenital Abnormalities and Anatomic Variants of the Pancreas (CAAVPs)

### Pancreas divisum

**Figure 2:**
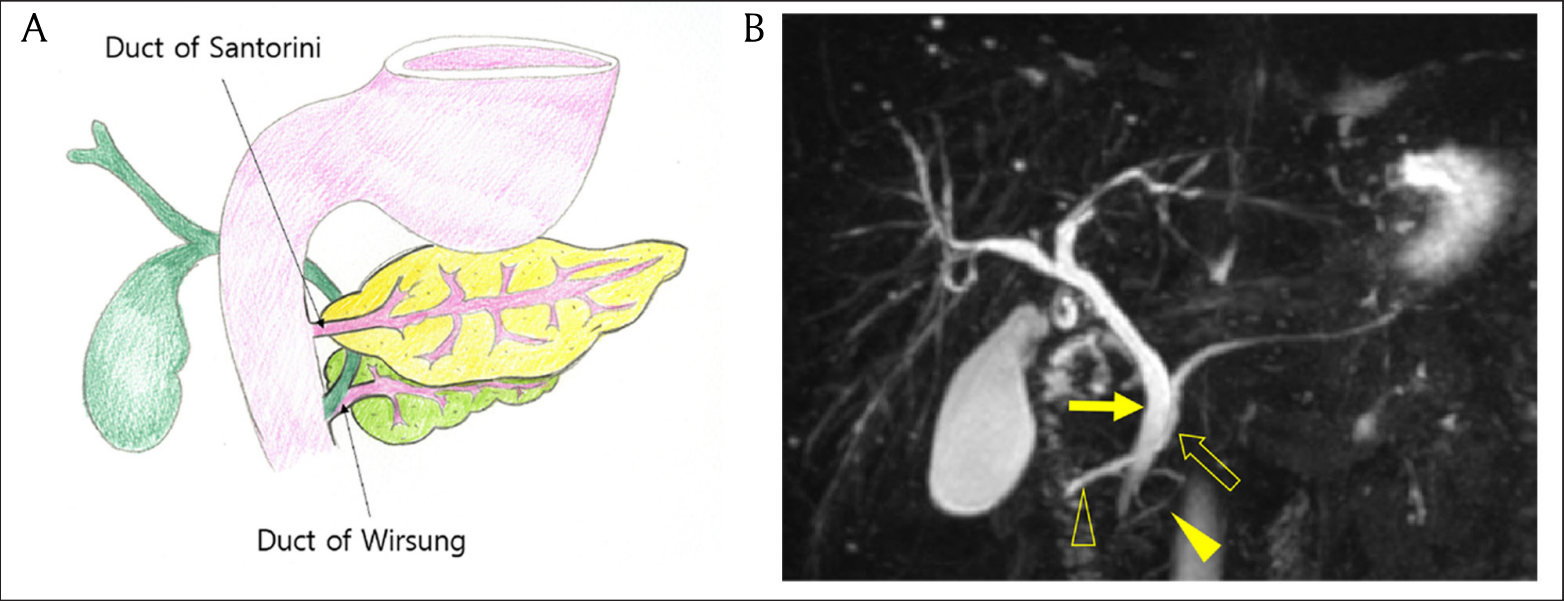
A 58-year-old woman with pancreas divisum. **(A)** Schematic shows failure to fuse ventral and dorsal pancreatic duct. **(B)** MRCP image shows crossed common bile duct (arrow) and the main pancreatic duct (open arrow), so called crossing duct sign. Main pancreatic duct is not fused with the ventral pancreatic duct (arrowhead) and continues with the duct of Santorini (open arrowhead).

**Figure 3:**
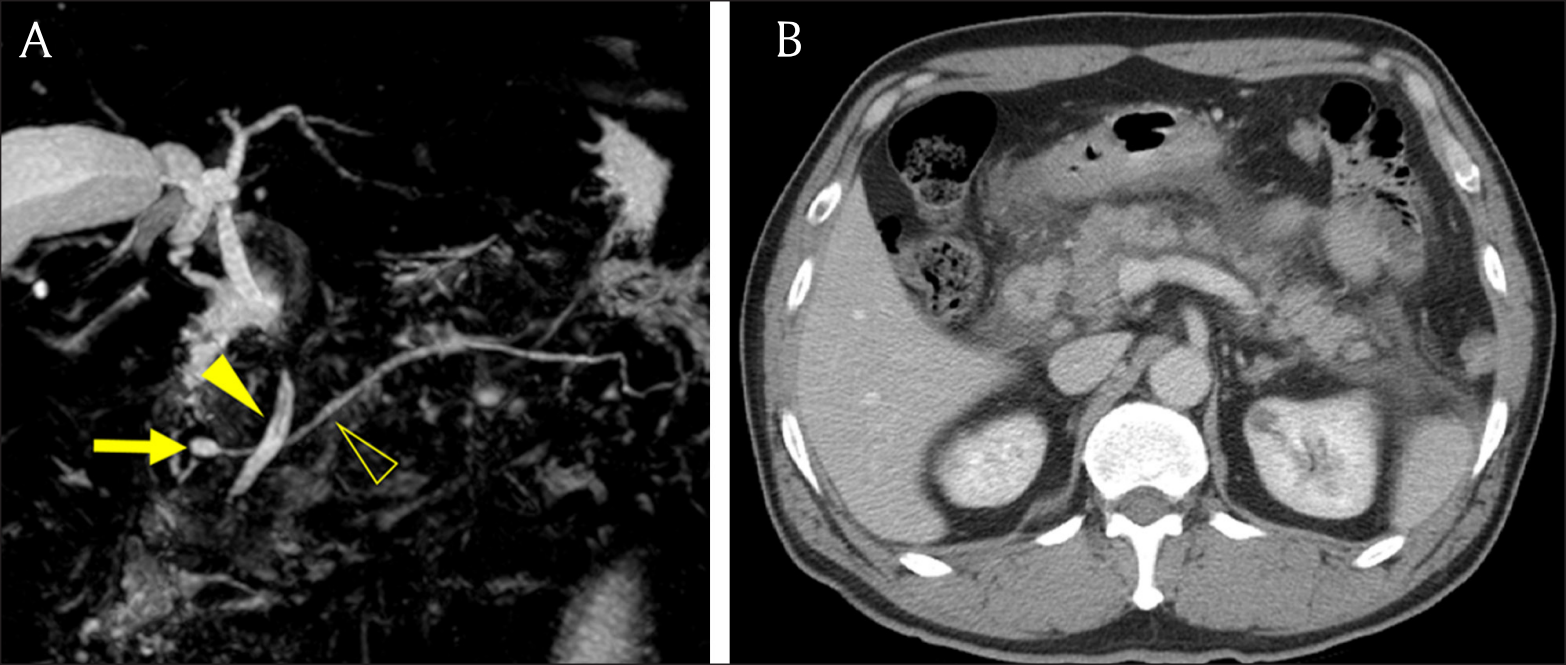
A 55-year-old man with pancreas divisum and Santorinicele who had history of recurrent pancreatitis. **(A)** MRCP image shows focal cystic dilatation of the distal portion of Santorini duct (arrow). Note crossed common bile duct (arrowhead) and main pancreatic duct (open arrowhead). **(B)** Axial portal venous phase CT image shows fluid collection around the pancreas, consistent with acute pancreatitis.

Pancreas divisum is the most common pancreatic congenital anomaly, and an autopsy series reported the prevalence as high as 14% [1]. This anomaly arises from failed fusion of the dorsal and ventral pancreatic ducts, resulting in two pancreatic ducts that are not joined together but separated and incompletely joined by very thin connections (Figure 2A). As a result, the dorsal pancreatic duct opens to the duodenum via the minor papilla. On MRCP, the common bile duct and the main pancreatic duct are crossed, the so-called ‘crossing duct sign’ (Figure 2B) [8]. In patients with pancreas divisum, exocrine secretory flow may produce through the duct of Santorini, more frequently resulting in recurrent pancreatitis. Some of them may have a Santorinicele (Figure 3) [1,2].

### Annular pancreas

**Figure 4:**
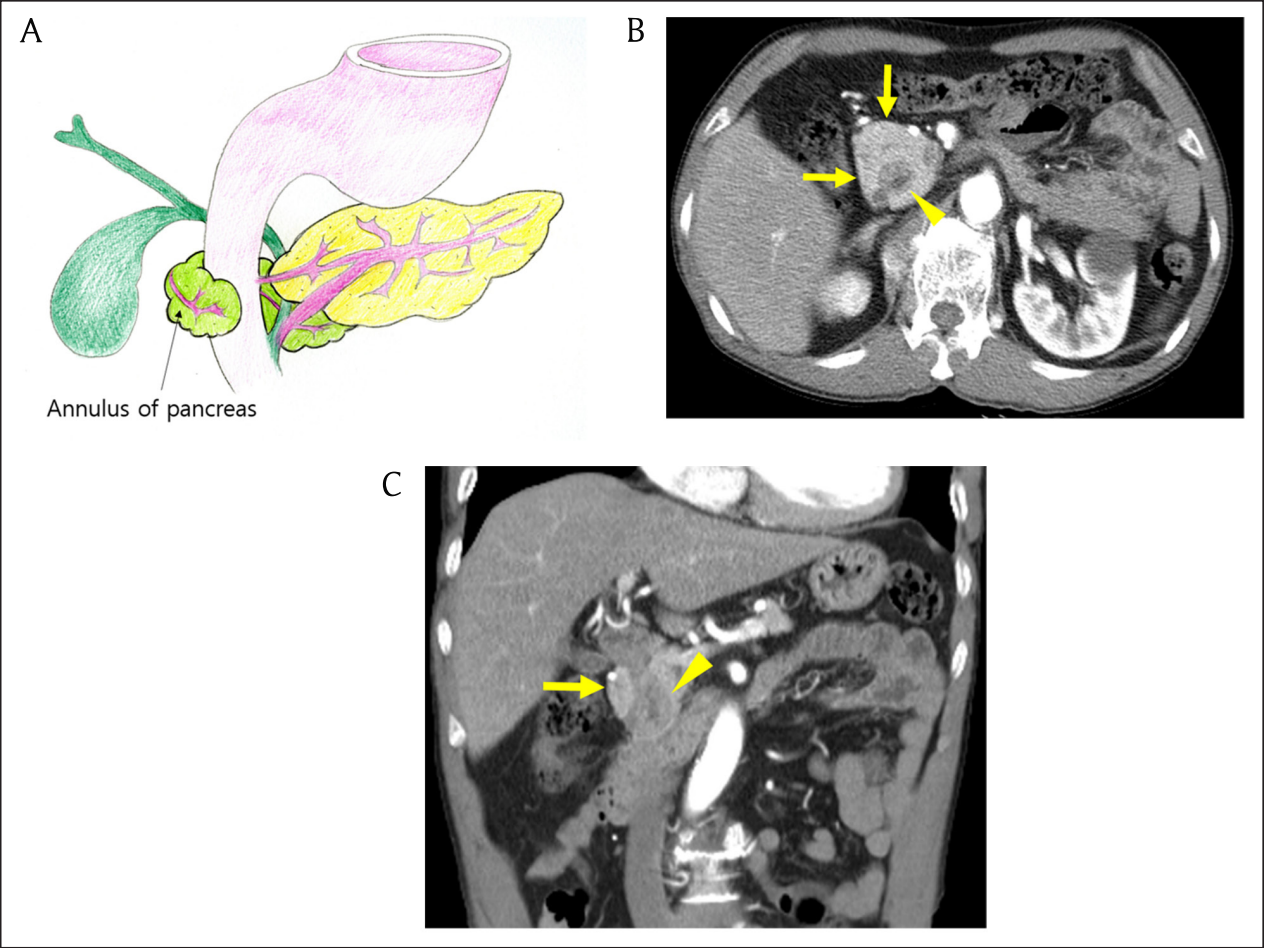
A 71-year-old man with annular pancreas. **(A)** Schematic shows the second part of duodenum surrounded by ventral pancreas. **(B, C)** Axial and coronal reformatted pancreatic phase CT images show pancreatic tissue (arrows) encircling descending portion of the duodenum (arrowhead).

**Figure 5:**
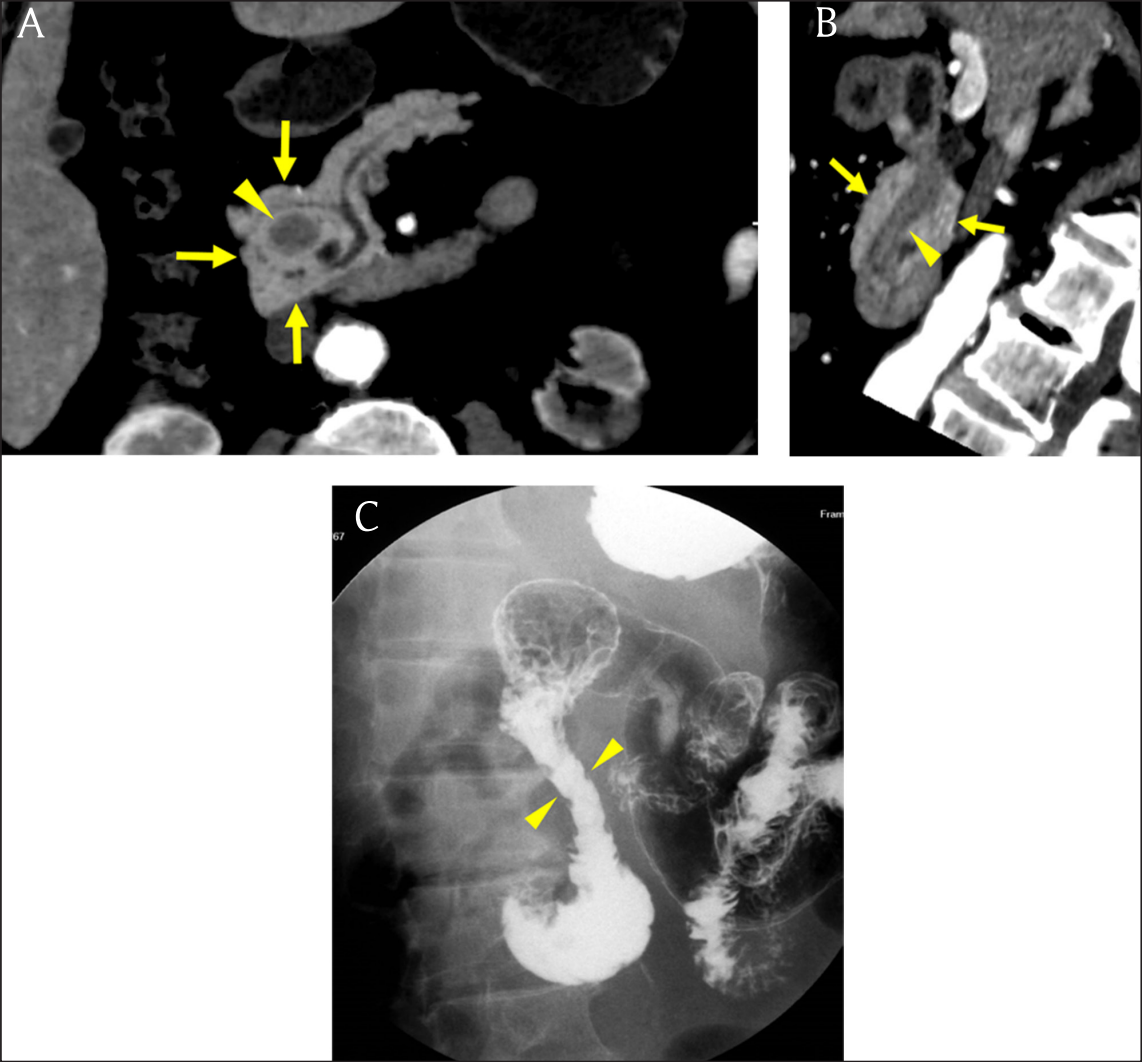
A 67-year-old man with annular pancreas. **(A, B)** Curved reformatted pancreatic phase CT images show pancreatic tissue (arrows) and duct around the second part of the duodenum (arrowhead). **(C)** Upper gastrointestinal series image shows concentric luminal narrowing of the descending duodenum (arrowheads).

Annular pancreas refers to as an anomaly in which the pancreatic tissue encircles the second portion of the duodenum [1]. The pancreatic tissue around the duodenum continues with the pancreatic head (Figure 4A) [2]. Annular pancreas is the second most common congenital anomaly of the pancreas, occurring in 1/20000 persons of the general population [2,9]. During embryonic development, the ventral pancreatic bud is composed of a right and left bud, and the left one normally disappeared. Annular pancreas is thought to be caused by adhesion of the right ventral bud to the duodenum or persistence of the left ventral bud [3,7]. The clinical manifestations of annular pancreas vary from congenital anomaly to malignancy [7,10]. Annular pancreas is easily detected on both CT and MRI (Figures 4 and 5) [1]. Surgical resection is needed in symptomatic patients [10].

### Dorsal pancreas agenesis

**Figure 6:**
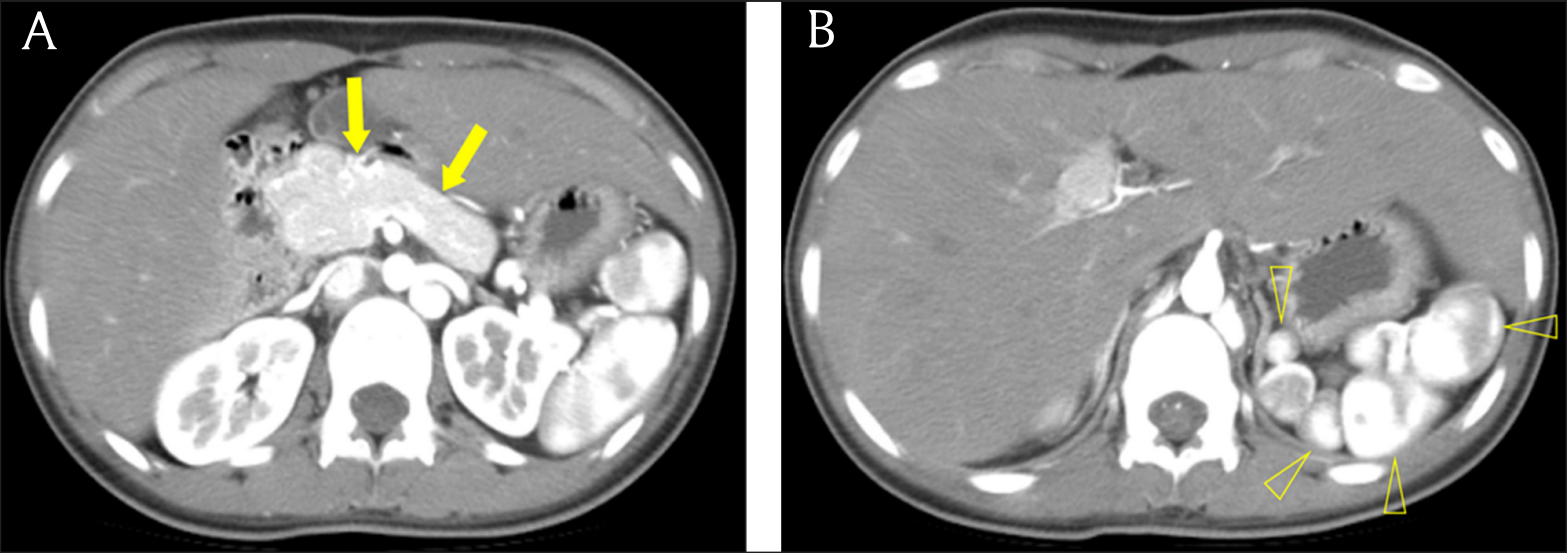
A 45-year-old man with heterotaxy syndrome. **(A)** Axial pancreatic phase CT image shows the short pancreas (arrows), suggesting partial agenesis of dorsal pancreas. **(B)** The multiple splenic tissues (open arrowheads) are located in the left side of the abdomen, indicating polysplenia.

**Figure 7:**
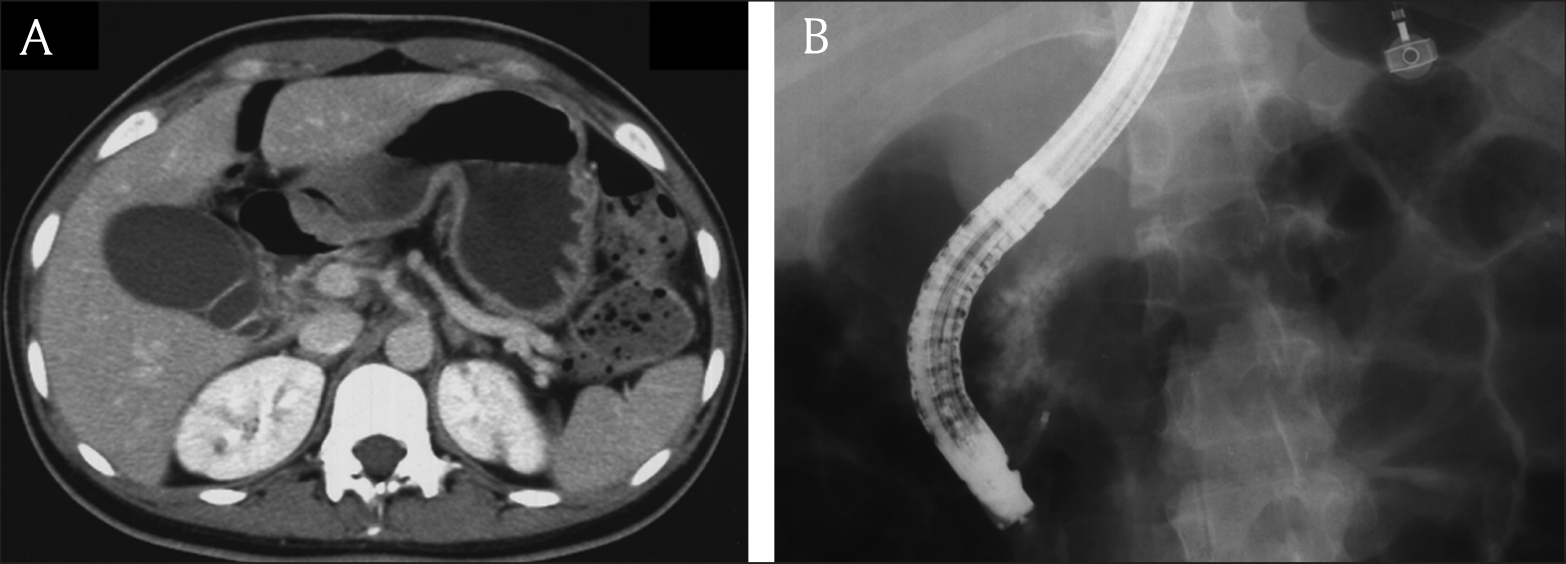
A 22-year-old woman with dorsal pancreas agenesis. **(A)** Axial portal venous phase CT image shows absence of dorsal pancreas. Note stomach in the distal pancreatic bed. **(B)** Endoscopic retrograde cholangiopancreatography image shows absence of dorsal pancreatic duct.

**Figure 8:**
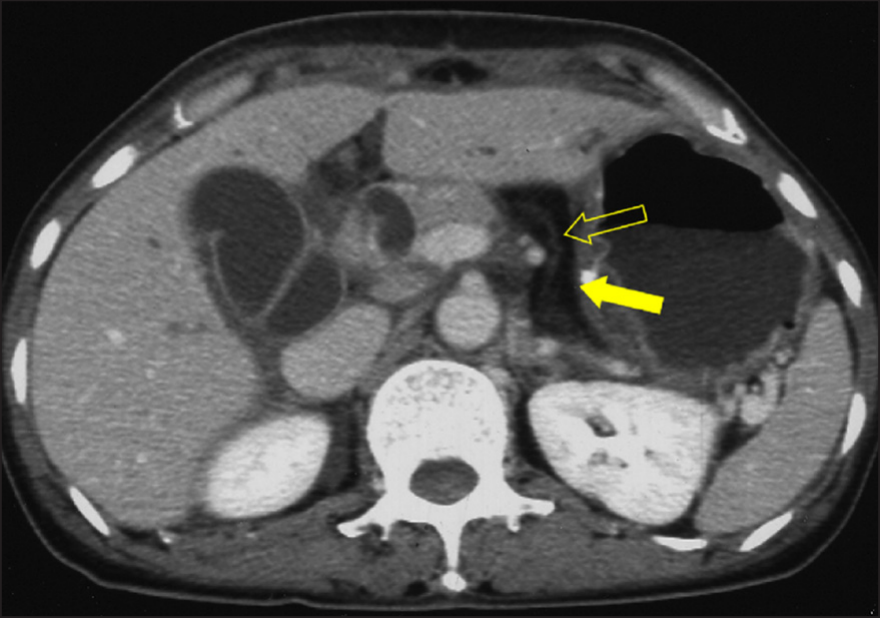
A 49-year-old woman with fatty replacement of distal pancreas. Axial portal venous phase CT image shows almost totally fatty change of distal pancreas (arrow). Note the pancreatic duct (open arrow) within the fat density pancreas tissue.

Dorsal pancreas agenesis is an uncommon anomaly that manifests as the absence of the body and tail of pancreas. This anomaly results from the absence of the dorsal pancreatic bud. Because most of the islet cells are located in the dorsal pancreas, dorsal pancreas agenesis is linked with diabetes mellitus [7]. Partial dorsal pancreas agenesis, also called short pancreas, is occasionally part of heterotaxy syndrome (Figure 6) [7]. It may be difficult to differentiate dorsal pancreas agenesis from fatty replacement of the distal pancreas, but the presence of stomach or intestine in the distal pancreatic bed and absence of the dorsal pancreatic duct favor a diagnosis of dorsal pancreas agenesis (Figures 7 and 8) [11].

### Heterotopic pancreas

**Figure 9:**
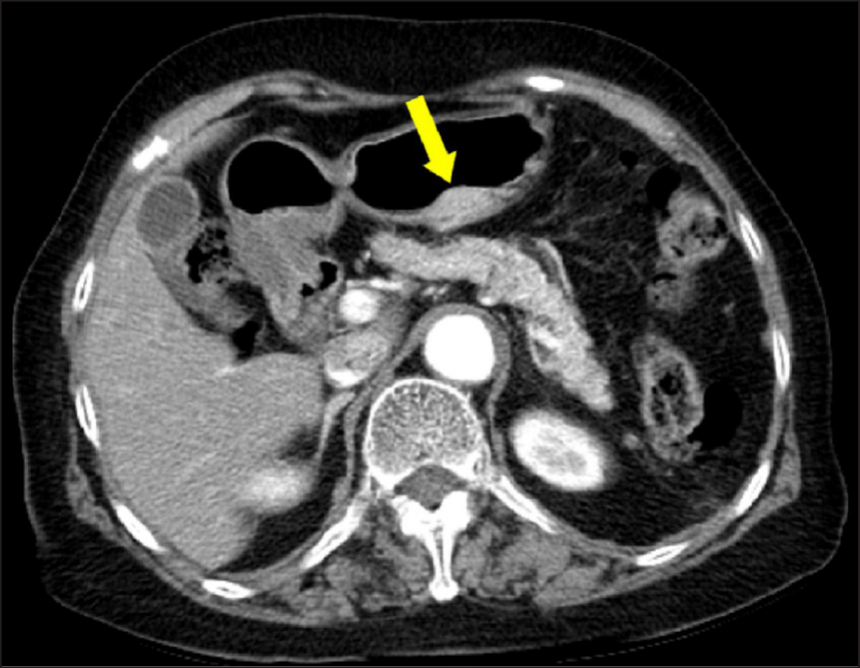
A 79-year-old woman with heterotopic pancreas in stomach. Axial pancreatic phase CT image shows an enhancing mass (arrow) in gastric antrum. The mass reveals ill-defined border and endophytic growth.

**Figure 10:**
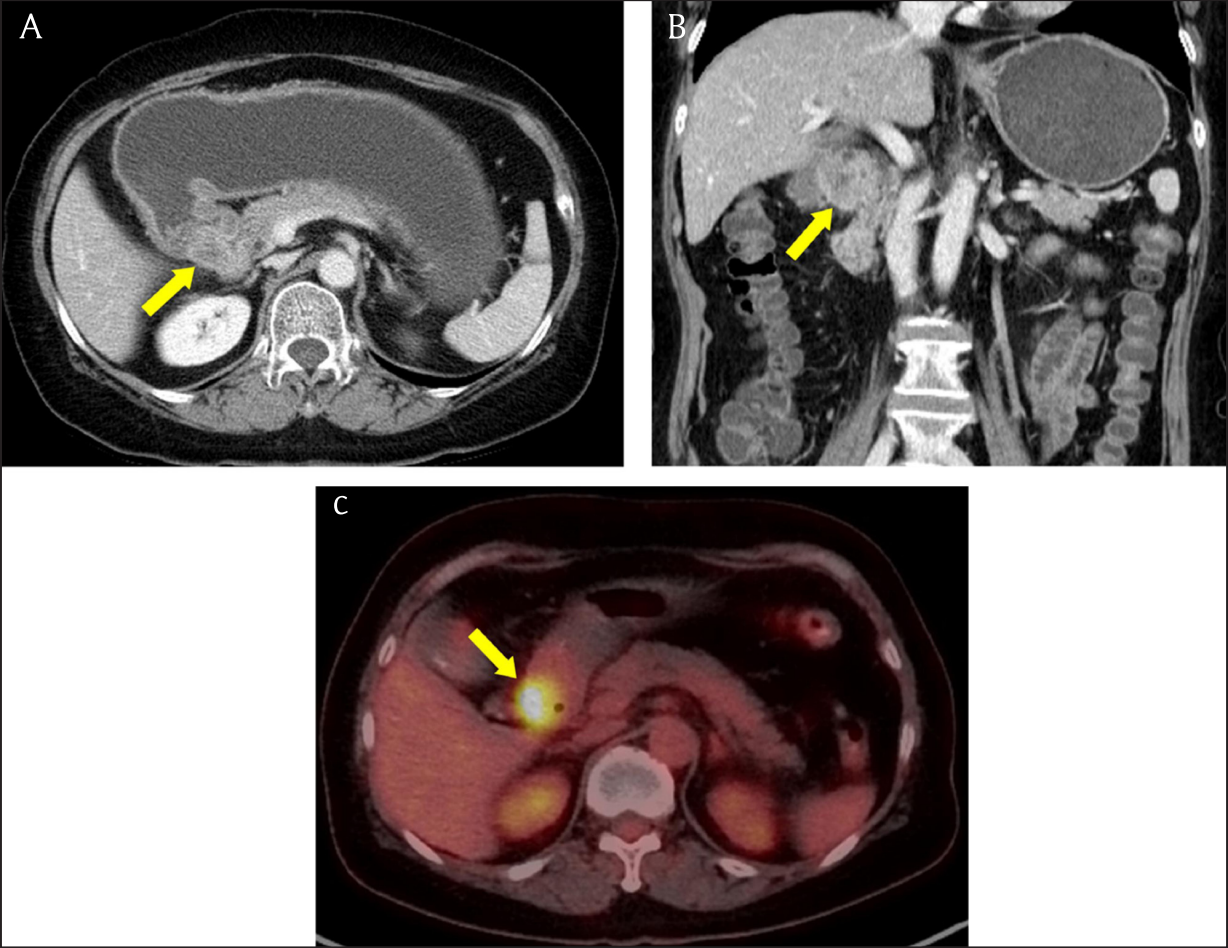
A 68-year-old woman with adenocarcinoma arising from heterotopic pancreas. **(A, B)** Axial and coronal reformatted CT images show irregular mass (arrow) with heterogeneous enhancement in gastric pylorus. **(C)** Positron emission tomography-CT image shows intense uptake in the mass (arrow).

**Figure 11:**
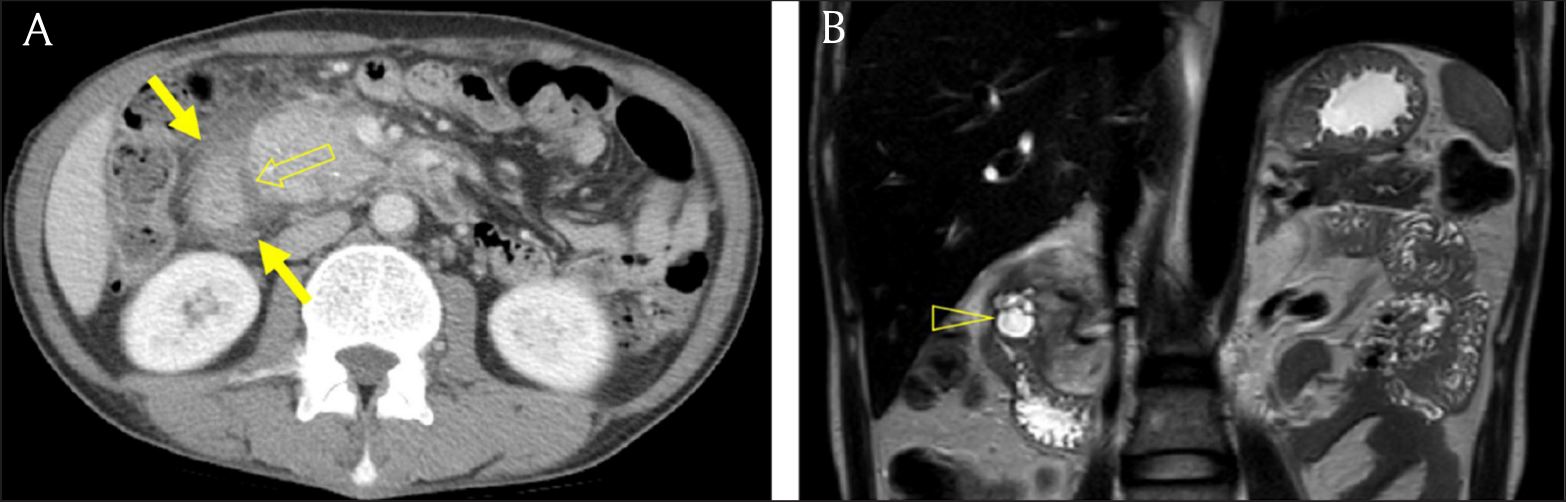
A 49-year-old man with paraduodenal pancreatitis. **(A)** Axial portal venous phase CT image shows fat infiltration and fluid collection (arrows) around the pancreaticoduodenal groove (open arrow). **(B)** Coronal T2-weighted image shows cystic lesion (open arrowhead) within the second portion of duodenum, suggesting cystic dystrophy of heterotopic pancreas.

Heterotopic pancreas occurs in 0.6%–13.7% of the general population and is defined as pancreatic tissue separated from the normal pancreas [12]. Although the aberration of the normal developmental process associated with heterotopic pancreas is unknown, the most widely accepted hypothesis is misplacement of pancreatic tissue [13]. The most common location of heterotopic pancreas is the proximal gastrointestinal (GI) tract. The stomach is concerned in 26–38%, the duodenum in 28–36%, and the jejunum in 16% [7]. On CT images, heterotopic pancreas usually measures less than 2 cm, and it can be misdiagnosed as a small submucosal tumor of the GI tract. Kim et al. [14] reported that CT findings useful for differentiation of heterotopic pancreas from gastrointestinal stromal tumor or leiomyoma in the stomach or duodenum are an ill-defined border, prominent enhancement of overlying mucosa, endoluminal growth pattern, prepyloric antrum or duodenal location, and flat shape (Figure 9). Malignant neoplasm or inflammation can develop from heterotopic pancreatic tissue (Figures 10 and 11) [7,12,15].

### Circumportal pancreas

**Figure 12:**
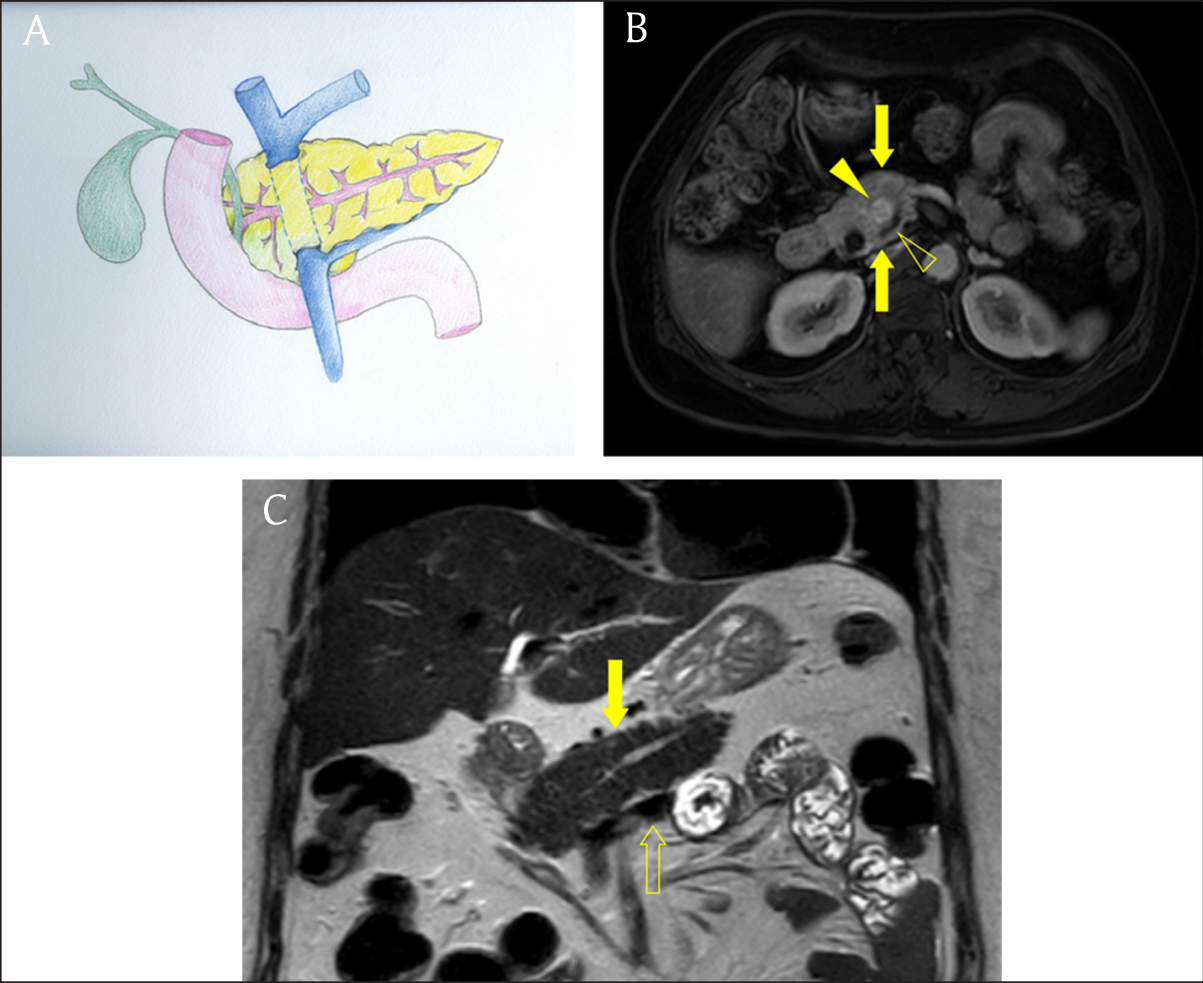
A 71-year-old man with retroportal suprasplenic type circumportal pancreas. **(A)** Schematic shows annulus of pancreatic tissue surrounding the portal vein. The main pancreatic duct passed behind the portal vein and pancreatic body and tail is located above the splenic vein. **(B)** Axial gadolinium-enhanced arterial phase MR image shows pancreatic tissue (arrows) encircling portal vein (arrowhead). Note the main pancreatic duct (open arrowhead) passing behind the portal vein. **(C)** Coronal T2-weighted image shows pancreatic body and tail (arrow) above the splenic vein (open arrow).

Pancreatic tissue can encase the portal vein or superior mesenteric vein (instead of the duodenum as in annular pancreas), and this anomaly is termed circumportal pancreas [16]. It is not rare and has a prevalence of 1.1% to 2.5% (Figure 12). Although the exact developmental mechanism of circumportal pancreas has not been elucidated, it is thought to result from abnormal fusion of the dorsal and ventral pancreatic buds [17]. Patients with this anomaly are usually asymptomatic, but it is clinically important to recognize circumportal pancreas prior to pancreatic surgery because its presence can change surgical planning or cause unexpected complications such as fistula formation [4,17]. Circumportal pancreas can be classified into the following four subtypes according to the course of the main pancreatic duct and the relationship between the splenic vein and the fused pancreas: 1) anteportal suprasplenic, 2) retroportal suprasplenic, 3) anteportal infrasplenic, and 4) retroportal infrasplenic [4].

### Intrapancreatic accessory spleen

**Figure 13:**
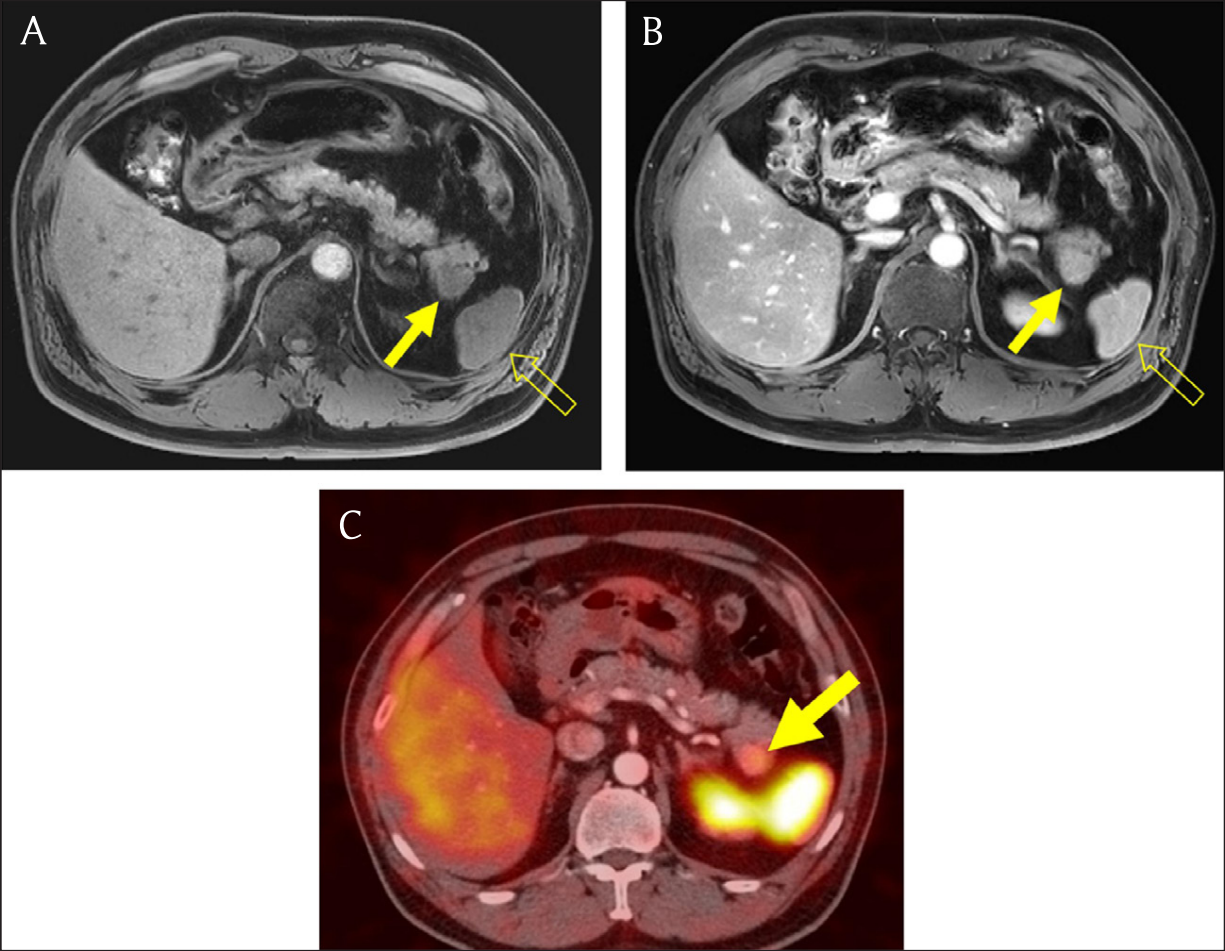
A 52-year-old man with accessory spleen in pancreatic tail. **(A)** Axial fat-suppressed T1-weighted image shows a mass-like lesion (arrow) with similar signal intensity compared with the spleen (open arrow) in pancreatic tail. **(B)** On axial gadolinium-enhanced portal venous phase MR image, the enhancement of the pancreatic mass (arrow) was similar to that of the spleen (open arrow). **(C)** Heat-damaged red blood cell scintigraphy shows intense uptake in the mass (arrow).

**Figure 14:**
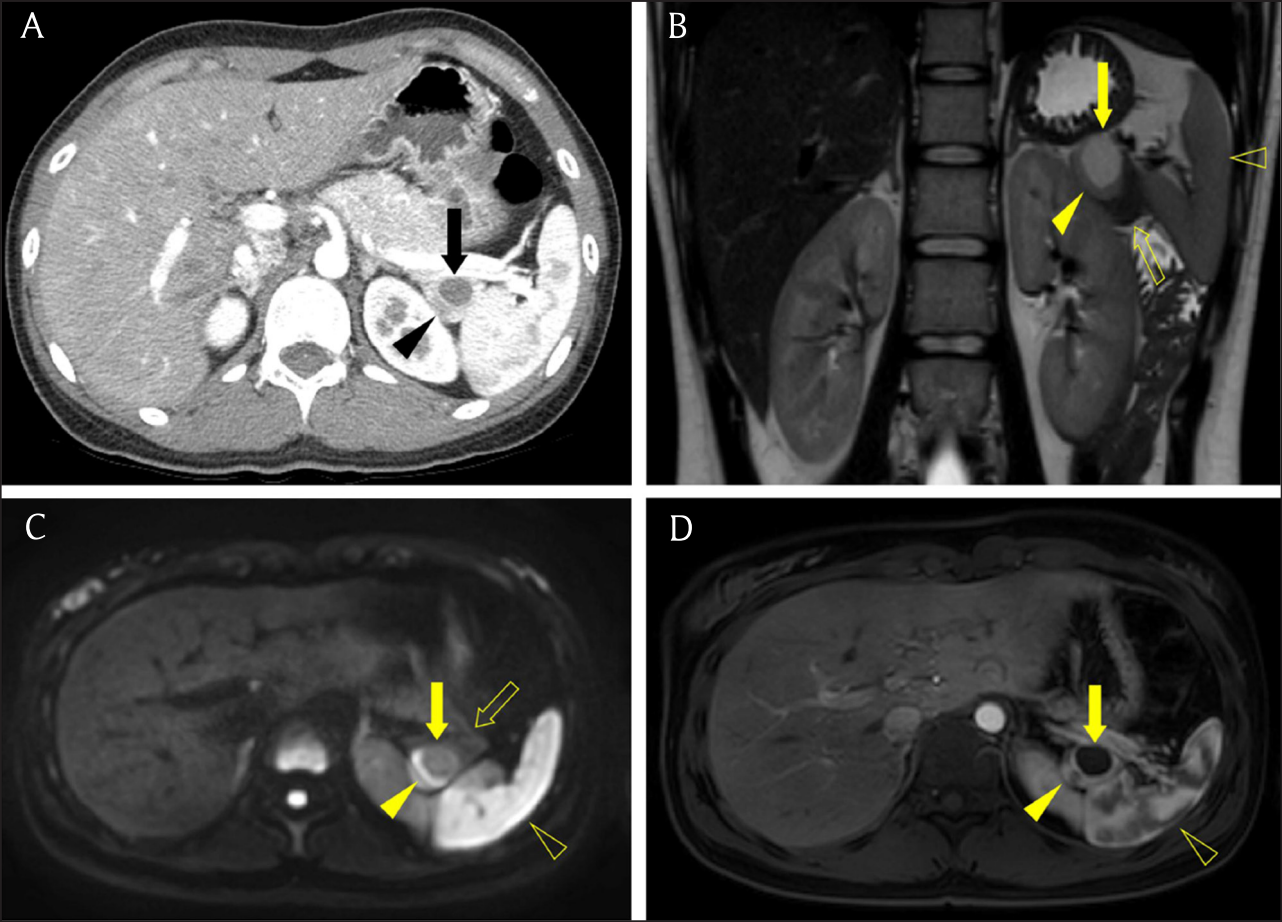
A 22-year-old woman with epithelial cyst arising from intrapancreatic accessory spleen. **(A)** Axial pancreatic phase CT image shows a 2.5 cm cystic lesion (arrow) with solid component (arrowhead) in the pancreatic tail. **(B, C)** MR images show that the signal intensity of the solid component (arrowhead) on T2-weighted image (B) and diffusion-weighted image (C) is closely similar to that of the spleen (open arrowhead) and different from that of the pancreatic tissue (open arrow). **(D)** Axial gadolinium-enhanced arterial phase MR image reveals zebra enhancement pattern of the solid component (arrowhead), like as spleen (open arrowhead). Note cystic portion (arrow) without enhancement.

An accessory spleen is an anatomical variation frequently observed in daily practice [18]. They result from failed fusion of the splenic precursors in the dorsal mesogastrium during the fifth week of embryogenesis [19]. Although most accessory spleens are located around the splenic hilum, approximately 16% are found in or around the pancreas [20]. An accessory spleen shows a similar signal intensity and enhancement compared with the mother spleen on MRI (Figure 13A and 13B) [19]. Radio-uptake on Tc-99m heat-damaged red blood cell scintigraphy can diagnose an accessory spleen without pathologic confirmation (Figure 13C) [19]. Rarely, an epithelial or epidermoid cyst can develop from an intrapancreatic accessory spleen (Figure 14) [19,21].

## Conclusion

CAAVPs are not uncommon. Many affected patients remain asymptomatic. However, some patients could present with symptom such as pancreatitis. In addition, CAAVPs can lead to unnecessary surgeries or unexpected complications. Familiarity with the imaging findings of various CAAVPs is paramount for managing patients in daily practice.

## Abbreviations

CAAVPs = Congenital abnormalities and anatomic variants of the pancreas

CT = Computed tomography

GI = Gastrointestinal

MRI = Magnetic resonance imaging

MRCP = Magnetic resonance cholangiopancreatography
